# Efficient whole-cell-catalyzing cellulose saccharification using engineered *Clostridium thermocellum*

**DOI:** 10.1186/s13068-017-0796-y

**Published:** 2017-05-12

**Authors:** Jie Zhang, Shiyue Liu, Renmin Li, Wei Hong, Yan Xiao, Yingang Feng, Qiu Cui, Ya-Jun Liu

**Affiliations:** 1 Shandong Provincial Key Laboratory of Energy Genetics, Qingdao Institute of Bioenergy and Bioprocess Technology, Chinese Academy of Sciences, Qingdao, 266101 People’s Republic of China; 2 CAS Key Laboratory of Biofuels, Qingdao Institute of Bioenergy and Bioprocess Technology, Chinese Academy of Sciences, Qingdao, 266101 People’s Republic of China; 3 Qingdao Engineering Laboratory of Single Cell Oil, Qingdao Institute of Bioenergy and Bioprocess Technology, Chinese Academy of Sciences, Qingdao, 266101 People’s Republic of China; 4 University of Chinese Academy of Sciences, Chinese Academy of Sciences, Beijing, 100049 People’s Republic of China; 5 Key Laboratory of Endemic and Ethnic Diseases (Guizhou Medical University), Ministry of Education, Guiyang, 550000 People’s Republic of China

**Keywords:** β-Glucosidase, Cellulosome, CelS, Fermentable sugar, Genome editing, Lignocellulose

## Abstract

**Background:**

Cost-efficient saccharification is one of the main bottlenecks for industrial lignocellulose conversion. *Clostridium thermocellum* naturally degrades lignocellulose efficiently using the cellulosome, a multiprotein supermolecular complex, and thus can be potentially used as a low-cost catalyst for lignocellulose saccharification. The industrial use of *C. thermocellum* is restrained due largely to the inhibition of the hydrolysate cellobiose to its cellulosome. Although the supplementation of beta-glucosidase may solve the problem, the production of the enzymes greatly complicates the process and may also increase the cost of saccharification.

**Results:**

To conquer the feedback inhibition and establish an efficient whole-cell catalyst for highly efficient cellulose saccharification, we constructed a recombinant strain of *C. thermocellum ∆pyrF*::*Ca*BglA which produced a secretory exoglucanase CelS-bearing heterologous BGL using a newly developed seamless genome editing system. Without the extra addition of enzymes, the relative saccharification level of *∆pyrF*::*Ca*BglA was stimulated by over twofolds compared to its parent strain *∆pyrF* through a two-stage saccharification process with 100 g/L Avicel as the carbon source. The production of reducing sugars and the relative saccharification level were further enhanced to 490 mM and 79.4%, respectively, with increased cell density.

**Conclusions:**

The high cellulose-degrading ability and sugar productivity suggested that the whole-cell-catalysis strategy for cellulose saccharification is promising, and the *C. thermocellum* strain *∆pyrF*::*Ca*BglA could be potentially used as an efficient whole-cell catalyst for industrial cellulose saccharification.

**Electronic supplementary material:**

The online version of this article (doi:10.1186/s13068-017-0796-y) contains supplementary material, which is available to authorized users.

## Background

Lignocellulosic biomass is the most abundantly available raw material on the Earth. Its sustainability and effective cost make it an attractive feedstock to substitute fossil resources [1–3]. Because of the recalcitrant structure, the main obstacle of lignocellulose bioconversion is the high cost of deconstruction [4–6]. *Clostridium thermocellum* has previously been demonstrated to have outstanding potential in lignocellulose bioconversion [7], because it produces a cellulosome, a multiprotein supermolecular complex, for highly efficient degradation of cellulose [8, 9]. By assembling various enzymatic subunits and multiple structural scaffoldings together, the cellulosome makes full use of the synergy effects resulting from the interactions to promote the lignocellulose hydrolysis process [10, 11]. Thus, the cellulosome-producing *C. thermocellum* is naturally suitable for lignocellulose bioconversion [12–14]. Nevertheless, the wild-type *C. thermocellum* cannot be directly used as a whole-cell industrial catalyst so far. One of the uppermost problems is the feedback inhibition caused by the end-product cellobiose to the cellulosome greatly limits the continuous cellulose saccharification [15, 16].

Currently, the feedback inhibition is generally relieved by supplying beta-glucosidases (BGL) into the hydrolysis system. For example, the targeted integration of BGL into the *C. thermocellum* cellulosome could enhance the degradation of cellulosic substrates [17]. Prawitwong et al. obtained high production of glucose using a *C. thermocellum* culture supplemented with a BGL from *Thermoanaerobacter brockii* [18]. Many efforts have been made to obtain low-cost BGLs, including the screening and engineering of BGLs for higher activity [19–23], and the development of novel processes for protein recycling [24, 25]. However, a relatively high load of the enzymes is still unavoidable because the activity and stability of the added BGLs are continuously decreasing during the hydrolysis process. In addition, the production and supplementation of BGL may greatly complicate the saccharification process.

To avoid the extra addition of enzymes in the saccharification system, we consider one of the most convenient and efficient way is to construct a recombinant *C. thermocellum* simultaneously producing secretory BGL as a whole-cell catalyst for cellulose saccharification. To relieve the feedback inhibition and promote the cellulose hydrolysis process, the expressed BGL should function synergistically with the cellulosome by assembling and form a substrate-coupled catalyzing pathway (cellulose–cellobiose–glucose) with other cellulosomal enzymes. The exoglucanase CelS (also known as Cel48S) is the most abundant enzymatic subunit in the cellulosome of *C. thermocellum* [26, 27], and a previous study supports it has the major contribution to cellulosome function [28]. Since CelS is the main producer of cellobiose in the cellulosome, relieving the cellobiose inhibition to CelS should be a priority. Thus, the fusion of BGL with CelS may be a preferred way for BGL supplementation. To achieve the fused expression of CelS with BGL in *C. thermocellum*, precise genome-editing methods, such as the markerless gene-deletion approach [29], are required.

## Results

### Selection of beta-glucosidases

Beta-glucosidases from *Caldicellulosiruptor* sp. F32 (*Ca*BglA), *C. thermocellum* DSM1313 (*Ct*BglA) and *T. brockii* DSM1457 (CglT), and a compost microbial metagenome (Td2f2) were preliminarily chosen as the candidates according to previous biochemical analyses [23, 25, 30–32], and were characterized under the optimal conditions of the cellulosome of *C. thermocellum* (pH 5.5 and 55 °C) for better coordination (Additional file 1). Under such reaction conditions, *Ca*BglA exhibited the highest specific activity (346.0 ± 3.7 U/mg) and thermal stability. *Ct*BglA had moderate activity (208.2 ± 4.8 U/mg) and sensitivity to glucose. CglT showed the highest glucose tolerance but relatively low specific activity (79.3 ± 5.9 U/mg). Td2f2 could be stimulated with the addition of glucose, but its specific activity (9.2 ± 0.3 U/mg) and thermal stability were low (Table 1; Additional file 1). Hence, *Ca*BglA and *Ct*BglA were chosen to express in *C. thermocellum* DSM 1313.

**Table 1:** Enzymatic properties of selected BGLs

Protein	Optimal temp (°C)/pH^a^	Specific activity (U/mg)	Thermal stability at 60/80 °C (%)^b^	Glucose inhibition (mM)^c^
*Ca*BglA	75–80/5.0–5.6 [23]	346.0 ± 3.7	99.3/91.4	314
*Ct*BglA	65/5.5	208.2 ± 4.8	116.9/4.8	205
CglT	60–75/6.0–7.0 [25]	79.3 ± 5.9	104.0/10.5	450
Td2f2	75/5.5 [31]	9.2 ± 0.3	71.1/40.9	None^d^

All experiments were performed in triplicate to calculate the averages and standard errors with *p*NPG as a substrate, and the reaction conditions were at pH 5.5 and 55 °C

^a^ The optimal temperature and pH of *Ca*BglA and *Ct*BglA were also determined in this study as shown in Additional file 1

^b^ The thermal stability was shown as percentage of remaining BGL activity after incubating at 60 or 80 °C for 24 h

^c^ The glucose inhibition of BGLs was determined by adding glucose at different concentrations (0–600 mM) to the standard reaction mixture, and calculated as the glucose concentration required to inhibit 50% of initial BGL activity (Additional file 1)

^d^ Instead of inhibition, Td2f2 could be stimulated by 29.2–72.0% with the addition of 100–600 mM glucose, which was consistent with previous report [31]

### Plasmid-dependent expression of BGLs in *C. thermocellum*

We initially tried to express BGLs in *C. thermocellum* using a replicating plasmid which is convenient to construct and to demonstrate the feasibility of BGL integration in cellulosome in vivo. pHK-*Ct*BglA-Doc and pHK-*Ca*BglA-Doc were constructed for the expression of fusion proteins *Ca*BglA-Doc and *Ct*BglA-Doc, respectively, under the control of the CelS promoter. The expressed proteins would contain a signal peptide for protein secretion and a dockerin (Doc) module for cellulosome assembly. Weak bands of ~65 kDa was detected by sodium dodecyl sulfate–polyacrylamide gel electrophoresis (SDS-PAGE) analysis of the cellulosomes (data not shown), but mass spectroscopy analysis failed to verify the fusion protein due to the low concentration. This result indicated very few, if any, BGL expression and assembly in the cellulosomes of the recombinant strains. BGL assay was then performed against *p*-nitrophenyl-β-d-glucopyranoside (*p*NPG) to confirm the assembly of the expressed BGLs to the cellulosomes and their functionality. 1.15 ± 0.07 U/mg BGL activity was detected in the cellulosome of *∆pyrF*::pHK-*Ca*BglA-Doc, but no BGL activity was detected in the cellulosome of *∆pyrF* and *∆pyrF*::pHK-*Ct*BglA-Doc, indicating the plasmid-based expression of the fusion protein *Ca*BglA-Doc with low abundance. The failed detection of *Ct*BglA-Doc indicated its failed expression or secretion. The difficulty of the plasmid-based expression in *C. thermocellum* has been discussed in previous studies [33, 34]. Nevertheless, the BGL activity of the cellulosome of *∆pyrF*::pHK-*Ca*BglA-Doc demonstrated that *C. thermocellum* can express and secrete active *Ca*BglA. Thus, we decided to introduce the BGL-encoding genes into *C. thermocellum* by chromosomal integration to relieve the metabolic burden that resulted from plasmid replication and the expression of plasmid-carrying antibiotic-resistant genes. In addition, the chromosomal gene expression is also more convenient for industrial application than the plasmid-based expression.

### Seamless knock-in of BGL gene in the genome of *C. thermocellum*

**Fig. 1:**
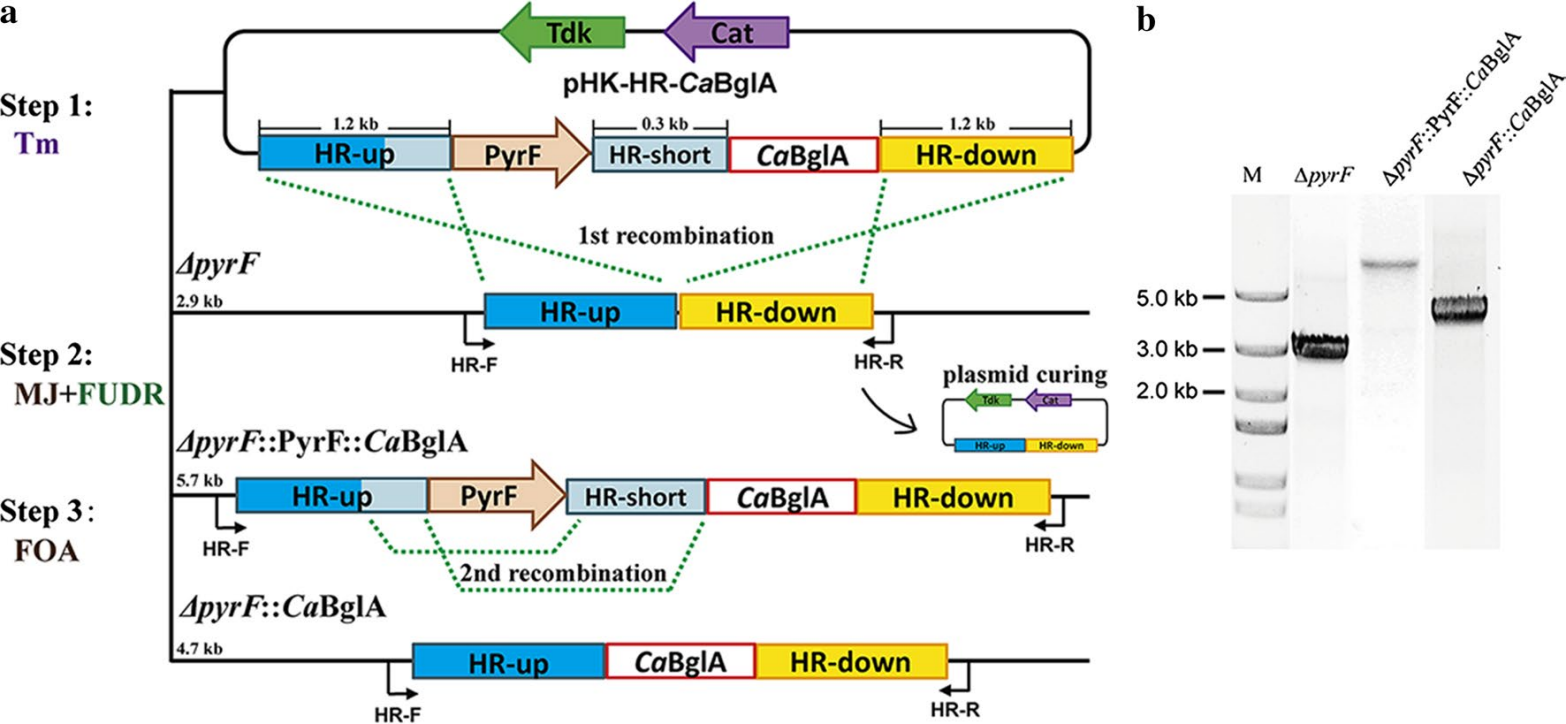
Knock-in of gene *ca*BglA in the chromosome of *C. thermocellum* ∆*pyrF.* **a** Schematic illustrating the work flow of the seamless genome editing system using plasmid pHK-HR-*Ca*BglA. Three screening steps including two rounds of recombination are involved. The first step consists of the transformation of plasmid into ∆*pyrF* strain, and the selection on Tm. The second step employs the combined selection of FUDR and uracil auxotrophic MJ medium to promote the integration of the “PyrF-HR-short-*Ca*BglA” fragment onto the chromosome and the elimination of the transformed plasmid. The PyrF function of the host cell was restored in this step. In the third step, the FOA selection stress promotes the removal of *pyrF* cassette through the second round of recombination. **b** Diagnostic PCR investigation of the obtained recombinant strain after each selection step. The target strain *∆pyrF*::*Ca*BglA shows a PCR product of 4.7 kb, indicating the successful knock-in of *caBglA* gene in the chromosome. *M* DNA marker

A seamless genome editing system was developed on the basis of the allele-coupled exchange (ACE) strategy [35], including a *pyrF*-deleted chassis strain ∆*pyrF* and corresponding plasmids for homologous recombination. The ACE strategy was originally developed in mesophilic clostridia, and it had not been used in the genetic engineering of thermophilic *C. thermocellum* previously according to our knowledge [35]. Three regions of homology with different lengths (two long regions of ~1.2 Kb and one short region of ~300 bp) were involved to control the order of homologous recombination events. Two selection markers, an orotidine 5-phosphate decarboxylase encoding gene *pyrF* from *C. thermocellum* DSM1313 and a thymidine kinase encoding gene *tdk* from a thermophilic anaerobe *Thermoanaerobacter* sp. X514 [36, 37], were used to achieve the markerless manipulation (Additional file 2). With the developed genome editing system, seamless gene deletion, insertion and replacement could be achieved after three screening steps and two rounds of recombination (Fig. 1).

We opted to create a BGL-CelS fusion protein to form a substrate-coupled catalyzing channel because removing the cellobiose produced by the exoglucanase CelS, the most abundant cellulosome subunit in *C. thermocellum,* might greatly release the feedback inhibition to the whole cellulosome system. BGL was designed to locate between the catalyzing module (Cel) and dockerin module (Doc) of CelS to avoid the interference to the type I dockerin–cohesin interaction during cellulosome assembly. The produced fusion protein Cel-BGL-Doc would contain three functional modules.

The plasmid pHK-HR-*Ca*BglA was constructed for the knock-in of gene *caBglA*. The termination codons of the BGL-encoding genes were eliminated during the plasmid construction to guarantee the fused expression with CelS modules. The recombinant strain *∆pyrF*::*Ca*BglA was obtained after three screening steps (Fig. 1a). In short, the *∆pyrF* transformants containing pHK-HR-*Ca*BglA were initially screened on solid GS-2 medium with Tm. Then, the recombinants were selected in MJ medium lacking uracil but containing FUDR. Under such selection stress, the plasmid containing *tdk* cassette must be cured to ensure the cell growth, while because the host cell was *pyrF*-deleted, the *pyrF* cassette on the plasmid was required to produce uracil. Thus, the first round recombination event occurred through two long regions of homology—HR-up and HR-down—to integrate both the selection marker *pyrF* and *Ca*BglA gene onto the chromosome. The *pyrF* function of the host strain was restored in this step, and the plasmid backbone was cured. In the third step, the counter-selection function of *pyrF* was used for the removal of *pyrF* cassette through the second round of recombination between the short region of homology HR-short and the 3′ region of HR-up, which harbored the same sequence with HR-short. The cells without PyrF function were selected in FOA-supplemented GS-2 medium. Colony PCRs and sequencing were performed using primer set HR-F/R to verify the recombination after each step of screening (Fig. 1b). We also tried to fuse the endogenous BGL of *C. thermocellum* DSM1313 (*Ct*BglA) with CelS. However, the knock-in of *ctBglA* gene was not successful even after several attempts. The construction got stuck during the first round of recombination in the second selection step (Fig. 1a), and no positive recombinant could be detected after screening hundreds of colonies. The difficulty might due to the preferred recombination occurred between the homologous *ctBglA* sequences, since the *ctBglA* sequence (~1.3 kb) was longer than the regions of homology (~1.2 kb).

### Investigation of BGL expression by *C. thermocellum*

**Fig. 2:**
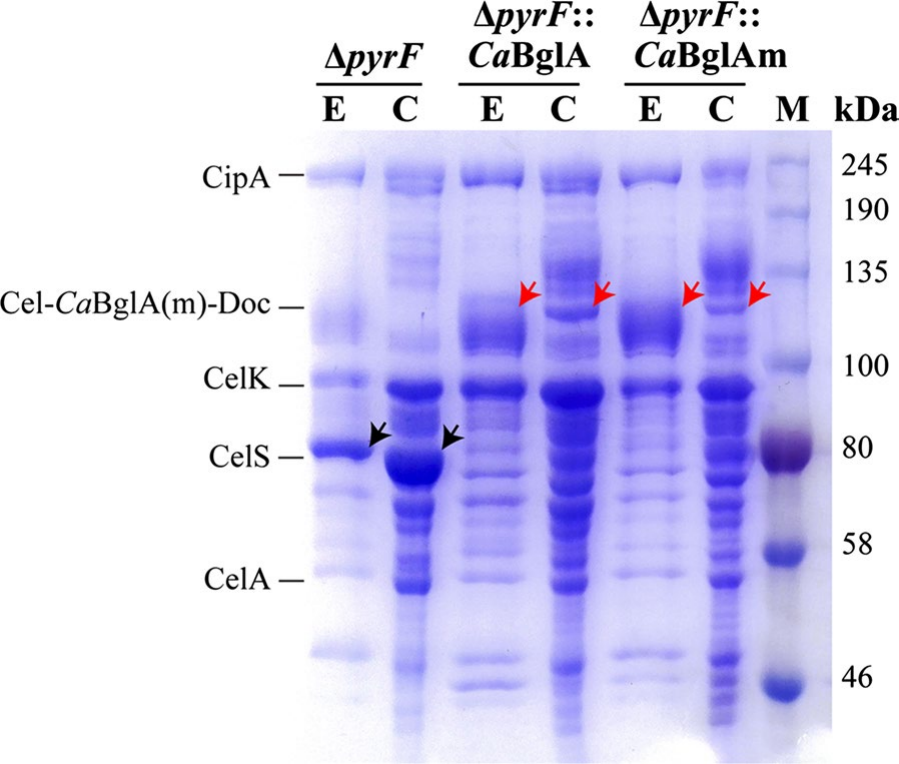
SDS-PAGE analysis of cellulosomal (*C*) and extracellular proteins (*E*) of *C. thermocellum* strains. The parent strain *∆pyrF* produced an intact CelS protein (*black arrows*). Compared to *∆pyrF,* an additional ~135-kDa band was observed in both cellulosomal and extracellular proteins of *∆pyrF*::*Ca*BglA and *∆pyrF*::*Ca*BglAm (*red arrows*), suggesting the successful expression of the fusion protein Cel-*Ca*BglA(m)-Doc, and its assembly in the cellulosome. Cellulosomal and extracellular proteins of *∆pyrF*::*Ca*BglA with the size of ~135 and ~75 kDa were further identified by mass spectroscopy (Additional file 3). Bands corresponding to known cellulosomal proteins are identified to the *left* of the Coomassie blue-stained gel. *M* protein standards

The cellulosomes and extracellular proteins of *∆pyrF*::*Ca*BglA were prepared and analyzed to confirm the expression of the fusion protein Cel-BGL-Doc with a theoretical size of ~135 kDa. The samples from the parent strain *∆pyrF* were also analyzed as the control. SDS-PAGE analysis showed that the ~75-kDa band referring to the wild-type CelS protein was rarely detected for *∆pyrF*::*Ca*BglA, but an additional band of ~135 kDa was detected (Fig. 2), indicating the expression of the fusion protein instead of the wild-type CelS. The ~135-kDa protein was further confirmed as Cel-*Ca*BglA-Doc by mass spectroscopy analysis (Additional file 3). Furthermore, 19.1 ± 1.2 U/mg BGL activity was detected in the cellulosome of *∆pyrF*::*Ca*BglA, which was 16 times higher than that of plasmid-based *Ca*BglA expression in *∆pyrF*::pHK-*Ca*BglA-Doc. These results indicated the successful expression, secretion and cellulosomal assembly of the active Cel-*Ca*BglA-Doc in *∆pyrF*::*Ca*BglA.

We observed lower abundance of the fused protein Cel-*Ca*BglA-Doc in ∆*pyrF*::*Ca*BglA compared to that of the wild-type CelS in ∆*pyrF*, which might influence the efficiency of cellulose degradation. In order to investigate whether the decreased protein expression was because of the unsuitable codon usage of *caBglA* gene, another strain ∆*pyrF*::*Ca*BglAm was constructed by replacing *Ca*BglA of ∆*pyrF*::*Ca*BglA with a codon-modified *Ca*BglAm. The expression and cellulosomal assembly of the CelS-bearing *Ca*BglAm in ∆*pyrF*::*Ca*BglAm was confirmed by enzyme assay, but the expression of the fusion protein was not significantly different from that of ∆*pyrF*::*Ca*BglA according to the SDS-PAGE analysis (Fig. 2).

### Enhanced cellulosomal activity by CelS-bearing BGL

**Fig. 3:**
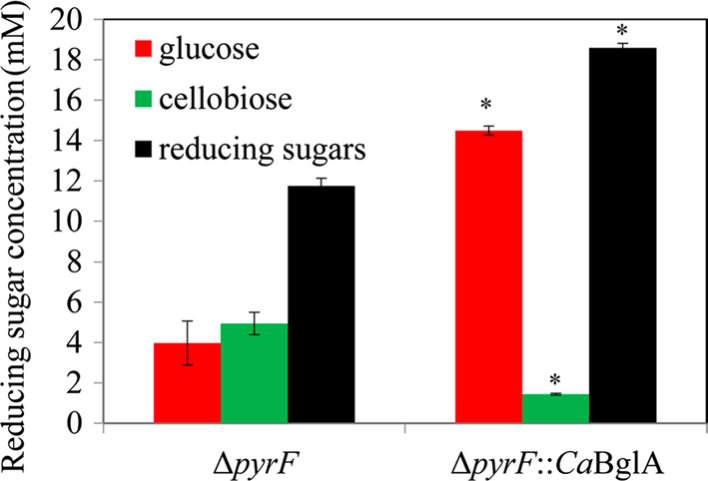
Cellulolytic activity of cellulosomes from *∆pyrF* and *∆pyrF*::*Ca*BglA. After 24-h hydrolysis assay at 55 °C with Avicel as the substrate, the degradation activity of cellulosomes was determined by quantifying the produced reducing sugar by DNS method and cellobiose and glucose by HPLC. Values are average ± standard deviation based on three independent replicates. *p < 0.01, *∆pyrF*::*Ca*BglA vs. *∆pyrF*

The cellulolytic activity of the cellulosome of *∆pyrF*::*Ca*BglA was analyzed by monitoring the concentrations of released reducing sugar after 24-h hydrolysis assay at 55 °C with Avicel as the substrate (Fig. 3). Cellobiose and glucose were also quantified by HPLC. The cellulosomal activity of *∆pyrF*::*Ca*BglA was 1.6-fold of the parent strain *∆pyrF*, while the glucose proportion in the released reducing sugar was increased from 34 to 78% due to the expression of *Ca*BglA, suggesting the enhanced cellulolytic activity. Although the cellulosome of *∆pyrF* contained no BGL activity against cellobiose, glucose was also detected in its hydrolysate against cellulose. This indicated that some endoglucanases involved in the cellulosome system of *C. thermocellum* could actively convert cello-oligosaccharides to glucose.

### Cellulose saccharification using *C. thermocellum* as a whole-cell catalyst

A two-stage process, including a cell-cultivation stage and a cellulose hydrolysis stage, was employed for cellulose saccharification. To determine the cultivation time in the two-stage process, the growth patterns of *∆pyrF* and *∆pyrF*::*Ca*BglA were initially analyzed using 5 g/L Avicel as a carbon source. The result showed that both strains grew in stationary phase after 28-h cultivation (Additional file 4). Thus, the cell-cultivation stage lasted for 36 h to guarantee the production of cellulosomal proteins and the complete utilization of the initial carbon source. In addition, *∆pyrF* and *∆pyrF*::*Ca*BglA showed similar Avicel consumption patterns (Additional file 4). This result indicated that the expression of CelS-bearing *Ca*BglA did not influence much the cellulose degradation of *C. thermocellum* at the cell-cultivation stage because the small amounts of accumulated sugars (2.24 ± 0.05 and 1.85 ± 0.85 mM cellobiose for *∆pyrF* and *∆pyrF*::*Ca*BglA, respectively; no glucose was detected) would show slight, if any, inhibition effect on cellulose hydrolysis [38].

**Fig. 4:**
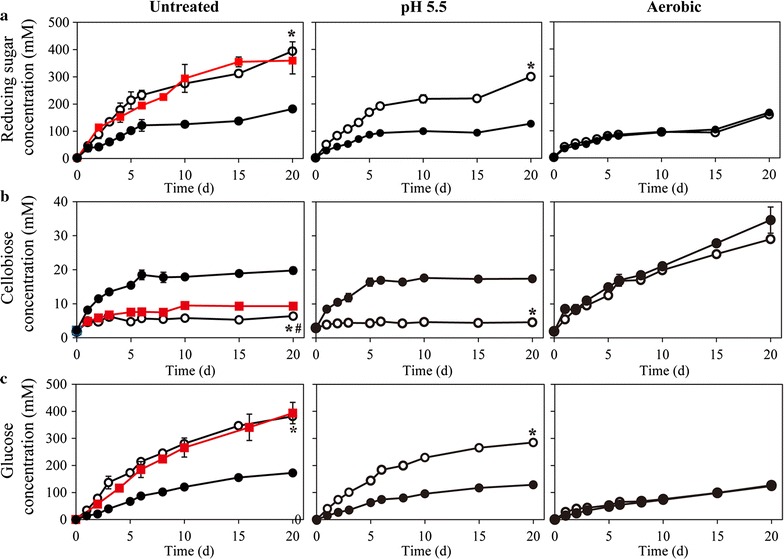
Production of total reducing sugar (**a**), cellobiose (**b**) and glucose (**c**) by *C. thermocellum* strains under various cellulose-saccharification conditions. Cells of both *∆pyrF* (*closed symbols*) and *∆pyrF*::*Ca*BglA (*open symbols*) were cultivated for 36 h in the cell-cultivation stage. For aerobic treatment (Aerobic), the cultures were transferred into 250-mL sterile flasks shaking at 170 rpm aerobically. For acidic treatment (pH 5.5), the pH value of the broths was adjusted to 5.5 by adding 1 N HCl in an anaerobic chamber. For BGL treatment (*red square*), 15 U/g cellulose of *Ca*BglA protein was added at the beginning of the saccharification process. Hydrolysis setups carried out under anaerobic condition without pH adjustment were used as the control (Untreated). 100 g/L Avicel was supplemented to initiate the cellulose hydrolysis under various conditions with different treatments. Values are average ± standard deviation based on three independent replicates. *p < 0.01, *∆pyrF*::*Ca*BglA vs. *∆pyrF* (*open* vs. *closed symbols*). #*p* < 0.01, *∆pyrF*::*Ca*BglA vs. *∆pyrF* with free *Ca*BglA (*open circle* vs. *red square*)

After the cell-cultivation stage, 100 g/L Avicel was supplemented to initiate the cellulose hydrolysis stage. *C. thermocellum* strains can assimilate the produced sugars [39], which may result in the decreased production of reducing sugars. As a strict anaerobe, the growth of *C. thermocellum* may cease with the presence of oxygen or low pH (pH value <6) [39]. Thus, we performed aerobic treatment (aeration) and acidic treatment (reduce the pH value to 5.5) to inhibit the cell growth as well as the assimilation process. Without any treatment*, ∆pyrF*::*Ca*BglA produced 394 ± 34.7 mM reducing sugar including 381 ± 13.3 mM glucose in 20 days. Meanwhile, the parent strain *∆pyrF* produced only 182 ± 8.7 mM reducing sugar including 173 ± 0.2 mM glucose. The results showed that *∆pyrF*::*Ca*BglA produced more reducing sugar than the parent strain under untreated as well as acidic conditions, but not under the aerobic condition (Fig. 4a). In addition, neither the aerobic nor acidic treatment was conducive to the production of reducing sugars by *C. thermocellum* strains, especially *∆pyrF*::*Ca*BglA (Fig. 4a). Under anaerobic (untreated or acidic) conditions, the parent strain ∆*pyrF* produced less glucose but accumulated more cellobiose than ∆*pyrF*::*Ca*BglA, indicating the expression of *Ca*BglA stimulated the conversion of cellobiose to glucose, thereby promoted the cellulose hydrolysis (Fig. 4b, c). However, *∆pyrF* and *∆pyrF*::*Ca*BglA produced similar amounts of glucose and cellobiose under aerobic condition (Fig. 4b, c). This suggested that the presence of oxygen inhibited not only the activity of cellulosome but also the activity of *Ca*BglA.

At the end of the cell-cultivation phase, the BGL activity in *∆pyrF*::*Ca*BglA broth was determined as 7.23 ± 0.2 U/g cellulose. Because there might be cell-bound CelS-bearing *Ca*BglA that did not release into the broth, we proposed higher BGL activity in *∆pyrF*::*Ca*BglA culture. To evaluate the contribution of expressed BGL to saccharification, a positive control was prepared by adding 15 U/g cellulose of purified *Ca*BglA protein in *∆pyrF* cultures at the beginning of the saccharification phase, and 359 ± 48.5 mM reducing sugar was produced after 20-day saccharification, which was similar to the production of *∆pyrF*::*Ca*BglA (Fig. 4a). The relative levels of cellulose saccharification from *∆pyrF*::*Ca*BglA and the positive control (*∆pyrF* supplemented with *Ca*BglA) were 63.9 and 58.2%, respectively. These results indicated the CelS-bearing *Ca*BglA in *∆pyrF*::*Ca*BglA was active as the free *Ca*BglA protein. In addition, although the glucose production showed no significant difference at the end of saccharification process, cellobiose concentration in the culture of the positive control was slightly higher than that in *∆pyrF*::*Ca*BglA culture (Fig. 4b, c), indicating more effective removal of cellobiose by CelS-bearing *Ca*BglA in *C. thermocellum*. These results suggested that the fusion of *Ca*BglA with CelS efficiently enhanced the cellulose saccharification. To investigate whether the production of reducing sugar could be further stimulated by increasing the activity of *Ca*BglA in *∆pyrF*::*Ca*BglA, 15 U/g cellulose of *Ca*BglA were also supplemented in the culture of *∆pyrF*::*Ca*BglA. However, we observed no obvious difference in the production of reducing sugars (Additional file 5).

The strain *∆pyrF*::pHK-*Ca*BglA-Doc producing plasmid-born *Ca*BglA-Doc protein was also used for cellulose saccharification, since its cellulosome contained the BGL activity. However, no increase of reducing sugar was detected in this strain compared with the parent strain, which might be explained by the low expression of *Ca*BglA. In addition, the saccharification activity of the strain ∆*pyrF*::*Ca*BglAm expressing a chromosome-born codon-modified *Ca*BglA was compared with ∆*pyrF*::*Ca*BglA, and no significant change was observed, either (Additional file 6).

### Improvement of the sugar production by *C. thermocellum ∆pyrF*::*Ca*BglA

Increased amount of whole-cell catalysts in the saccharification system might result in enhanced cellulose degradation and fermentable sugar production. Thus, we tried to stimulate the cell growth of *C. thermocellum ∆pyrF*::*Ca*BglA by modifying the fermentation medium. Cellobiose and ammonium sulfate were supplemented as extra carbon and inorganic nitrogen source, respectively [40]. Over 4.5-fold increase of the cell density (OD_600nm_ = 5.5) was observed when 20 g/L cellobiose and 1.3 g/L ammonium sulfate was used (Additional file 7). However, *∆pyrF*::*Ca*BglA only produced 241 ± 3.4 mM reducing sugar using such rich medium after 15-day saccharification, which was only 59% of that in the control setup using regular medium. This indicated that although *C. thermocellum* prefers cellobiose to cellulose and grows fast with increased carbon and nitrogen loading, the cellulosome produced using cellobiose as the carbon source may not be suitable for cellulose degradation due to the substrate-coupled regulation mechanism [41].

**Fig. 5:**
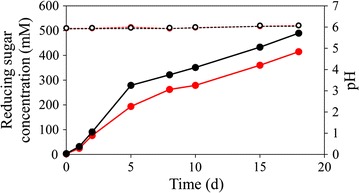
Saccharification and pH curves of *C. thermocellum* strain *∆pyrF*::*Ca*BglA with increased cell density. Cells from 200- to 300-mL culture were concentrated, resuspended, and reinoculated into 100 mL fresh GS-2 medium with 100 g/L Avicel as a sole carbon source to increase the cell density by 1.6 (*red*) or 2.4 fold (*black*). The concentration of produced reducing sugars (*closed circles*) and the pH values (*open circles*) were determined based on three independent replicates, and values are shown as average ± standard deviation

To avoid the potential impact of substrate change on the composition and activity of the cellulosome, concentrated *∆pyrF*::*Ca*BglA cells were inoculated in fresh medium, and the cell density was increased by 1.6- or 2.4-fold (the concentration of pellet protein increased from 0.26 to 0.46 or 0.63 mg/mL, respectively). The production of the reducing sugar was stimulated up to 490 ± 7.6 mM (Fig. 5), including 451 ± 5.7 mM (81.1 ± 1.0 g/L) glucose and 6.8 ± 0.4 mM (2.3 ± 0.1 g/L) cellobiose, and the saccharification increased to 79.4%. The pH values of the cultures were buffered and maintained at around 6 (Fig. 5). At the end of saccharification process, we observed complete degradation of the initial Avicel but no significant cell growth was detected (Additional file 8). Thus, the change of cell biomass would show slight influence on the carbon recovery. To investigate the carbon flux in the saccharification system in addition to fermentable sugars, we quantified potential intermediates and end metabolites including pyruvate, ethanol, lactate, acetate, and formate produced by ∆*pyrF*::*Ca*BglA. At the end of saccharification, ∆*pyrF*::*Ca*BglA with 2.4-fold cell density produced 261 ± 10.3 mM of ethanol, while trace amount of other products were detected. The produced ethanol was equivalent to around 131 mM glucose. Together, 620.5 ± 9.2 mM products in glucose equivalent were produced from the initial Avicel (617 mM in glucose equivalent), and the carbon recovery was 100.6 ± 1.6%.

## Discussion

*Clostridium thermocellum* can be used as a potential whole-cell catalyst for cellulose saccharification because of its robust cellulosome system [9], but proper engineering is indispensable to overcome its natural deficiencies, e.g. cellobiose inhibition, based on the industrial standard [5]. BGL supplementation is one of the most efficient strategies to relieve the cellobiose inhibition to cellulosome by previous in vitro studies [17]. Prawitwong et al. developed a biological saccharification system using a *C. thermocellum* culture supplemented with BGL to produce glucose [18], and the BGL recycling was considered by fusing BGL with a cellulose binding module [24, 25], but the additional production and supplementation of BGL complicated the process and may also increase the saccharification cost. In addition, the decreasing stability and the glucose inhibition to BGL in company with the saccharification process might require relatively high or successive load of BGL proteins, which may further increase the cost. In this study, we employed the whole-cell catalyzing strategy for lignocellulose saccharification without supplementation of extra enzymes. This strategy fits to the industry requirement of low-cost, simple process, and high efficiency, but needs an efficient whole-cell catalyst producing secretory BGL.

It is known that CelS plays a key role in cellulose hydrolysis by cellulosome and is the main producer of cellobiose [28, 42], thus the fusion of selected BGL with CelS would greatly release the feedback inhibition effect on cellulosome. By developing a novel ACE-based seamless genome editing system in the thermophilic bacterium, we successfully inserted a BGL gene *caBglA* into the CelS-encoding sequence of *C. thermocellum* DSM 1313 precisely between its catalyzing module and dockerin module. A recombinant strain *∆pyrF*::*Ca*BglA was finally constructed producing a CelS-bearing *Ca*BglA protein as an active cellulosomal component. Nevertheless, we observed decreased expression of the fused protein Cel-*Ca*BglA-Doc in ∆*pyrF*::*Ca*BglA compared to that of the wild-type CelS in ∆*pyrF*, which might influence the efficiency of cellulose degradation and sugar production. We thought the unsuitable codon usage of *caBglA* gene might be the problem, and optimized the codon of *caBglA* gene, but the result suggested that it was not the case. The fusion protein Cel-*Ca*BglA-Doc was expressed under the control of the strong promoter of CelS in ∆*pyrF*::*Ca*BglA, which might result in inherent resource shortage and therefore induce down-regulation of the expression level. Further studies are needed to reveal the reason of the low expression of the fused protein. Difference strategies to introduce BGL into cellulosome, such as fusion with other cellulosomal component, or fusion at different position of CelS, are also worth trying in future.

In addition to cellulosomal integration, we also achieved the plasmid-dependent expression of *Ca*BglA in *C. thermocellum* using a multicopy-replicating plasmid pHK. Although *∆pyrF*::pHK-*Ca*BglA-Doc theoretically contained more *caBglA* gene copies than *∆pyrF*::*Ca*BglA, and both employed the CelS promoter to drive the transcription, the cellulosome of *∆pyrF*::pHK-*Ca*BglA-Doc showed 16-fold lower BGL activity (1.15 ± 0.07 U/mg) than that of *∆pyrF*::*Ca*BglA (19.1 ± 1.2 U/mg). This result suggested episomal plasmid DNA might not be the proper vector for protein expression, and chromosomal integration might result in higher level of protein expression in some cases [43], and the CelS-bearing expression pattern might play key roles during the protein secretion and/or assembly process to support the functional expression of the heterologous BGL. This phenomenon should be considered when expressing other exogenous proteins in *C. thermocellum*.

Because the assimilation of cellodextrins by *C. thermocellum* may result in the decreased production of reducing sugars [39], we set up aerobic or low-pH treatments to inhibit the cell metabolism at the cellulose hydrolysis stage. However, neither aeration nor pH reduction resulted in increased production of reducing sugars. In contrast, lower sugar concentrations were detected compared with the untreated control. The negative effect of aeration on the cellulose hydrolysis might be explained by the oxygen sensitivity of the cellulosome [44, 45], and *Ca*BglA might also prefer anaerobic condition. Although pH 5.5 was determined as the optimal pH of both cellulosome [45, 46] and *Ca*BglA [23] (Additional file 1), low-pH condition decreased the production of reducing sugars in this study. We also detected decreased pellet protein and extracellular protein concentrations when the pH value of *∆pyrF*::*Ca*BglA was decreased to 5.5 at the beginning of the cellulose hydrolysis stage (Additional file 8). These results indicated that the low-pH condition might inhibit the cell growth as well as the continuous production of cellulosomal proteins by cells, and cell lysis might also occur under acidic conditions [39]. In addition, there might be other extracellular protein components that contributed to the cellulose hydrolysis but were not in favor of acidic conditions. Thus, the control of pH value should be considered in further application of *C. thermocellum* for cellulose conversion.

Although with addition of 15 U/g cellulose of purified *Ca*BglA protein, the parent strain *∆pyrF* showed similar saccharification activity to that of *∆pyrF*::*Ca*BglA, it accumulated more cellobiose during hydrolysis. This result indicated CelS-bearing *Ca*BglA expressed by *C. thermocellum* was more effective in cellobiose conversion than supplemented free protein. In contrast to the parent strain *∆pyrF*, further supplementation of purified *Ca*BglA in the culture of ∆*pyrF*::*Ca*BglA showed no apparent effect on sugar production, suggesting that the fused expression of Cel-*Ca*BglA-Doc led to the full occupation of CelS by *Ca*BglA. The formed substrate-coupled catalyzing channel had relieved most of the cellobiose inhibition effect on the whole cellulosome system. Under our whole-cell-catalyzing conditions, the carbon recovery of supplemented cellulose was about 100%, i.e., 79% of supplemented carbon was converted to soluble sugars and 21% to the end product ethanol. The productivity of fermentable sugars might be further enhanced by disrupting the metabolic pathways leading to end products, especially ethanol, in the recombinant strain *∆pyrF*::*Ca*BglA.

## Conclusion

Lignocellulosic biomass is an attractive feedstock to substitute fossil resources, but is difficult to deconstruct. *C. thermocellum* naturally degrades lignocellulose efficiently but cannot be directly used in industry due largely to the feedback inhibition to its enzymatic system cellulosome. The supplementation of BGL could solve the problem. However, the addition of enzymes produced elsewhere complicates the process and hinders the utilization of lignocellulose in industry. Here, we constructed an efficient whole-cell catalyst producing BGL for cellulose saccharification by targeted engineering of *C. thermocellum*. Without supplementation of any other enzymes, the whole-cell catalyst showed the high cellulose-saccharification activity and sugar production. Hence, our work confirmed the feasibility of the whole-cell-catalysis strategy for cellulose saccharification, and provided a potential whole-cell catalyst for industrial cellulose saccharification.

## Methods

### Bacterial strains and cultivation

The bacterial strains used in this study are listed in Table 2. *Escherichia coli* strains were cultivated aerobically at 37 °C in Luria–Bertani (LB) liquid medium with shaking at 160 rpm or on solid LB plate with 1.5% agar. *C. thermocellum* strains were grown anaerobically at 55 °C in modified GS-2 [47] or MJ medium [48] with 5 g/L cellobiose, 5 to 100 g/L Avicel (PH-101, Sigma) unless otherwise stated. 30 μg/mL chloramphenicol, 50 μg/mL kanamycin and 3 μg/mL thiamphenicol (Tm) were supplemented to the medium when necessary. 10 μg/mL 5-fluoro-2-deoxyuradine (FUDR) or 500 μg/mL 5-fluoroorotic acid (FOA) dissolved in dimethyl sulfoxide were added for screening.

**Table 2:** Bacterial strains and plasmids used in this study

Strains/plasmids	Relevant characteristic	Sources
Strains		
*E. coli*		
DH5α	*f80dlacZ*Δ*M15,* Δ*(lacZYA*-*argF)U169, deoR, recA1, endA1, hsdR17(r*_*k*_^−^ *, m*_*k*_^+^ *), phoA, supE44, l* ^−^ *, thi*-*1, gyrA96, relA1*	Transgen
BL21(DE3)	*ompT gal dcm lon hsdS*_*B*_ *(r*_*B*_^−^ *m*_*B*_^−^ *) l (DE3 [lacI lacUV5*-*T7 gene 1 ind1 sam7 nin5])*	Transgen
*C. thermocellum*		
DSM1313	LQ8, wild type stain	DSMZ
Δ*pyrF*	Derived from DSM1313, with deleted *pyrF* gene	This work
Δ*pyrF*::*Ca*BglA	Derived from Δ*pyrF*, expressing CelS-bearing *Ca*BglA	This work
Δ*pyrF*::*Ca*BglAm	Derived from Δ*pyrF*, expressing CelS-bearing *Ca*BglAm	This work
*∆pyrF*::pHK-*Ct*BglA-Doc	Derived from Δ*pyrF*, containing plasmid pHK-*Ct*BglA-Doc	This work
*∆pyrF*::pHK-*Ca*BglA-Doc	Derived from Δ*pyrF*, containing plasmid pHK-*Ca*BglA-Doc	This work
Plasmids		
pHK	pNW33N derivative, *E. coli*–*C. thermocellum* shuttle vector, Cm^R^/Tm^R^	[51]
pHK-∆pyrF	pHK derivative, containing upstream and downstream regions of *pyrF* for *pyrF* deletion	This work
pHK-HR	pHK derivative, containing *pyrF* cassette, *tdk* cassette, three regions of homology, and the target DNA sequence to knock in	This work
pHK-HR-*Ca*BglA	pHK-HR derivative for markerless knock-in of *caBglA* in the chromosome of DSM 1313	This work
pHK-HR-*Ct*BglA	pHK-HR derivative for markerless knock-in of *ctBglA* in the chromosome of DSM 1313	This work
pHK-HR-*Ca*BglAm	pHK-HR derivative for markerless knock-in of *caBglAm* in the chromosome of DSM 1313	This work
pHK-*Ct*BglA-Doc	pHK-HR-*Ct*BglA derivative for expression of *Ct*BglA-Doc in DSM 1313, with the promoter and signal peptide region of CelS	This work
pHK-*Ca*BglA-Doc	pHK-HR-*Ca*BglA derivative for expression of *Ca*BglA-Doc in DSM 1313, with the promoter and signal peptide region of CelS	This work
pET28aNS	Expression vector with N-terminal hexahistidine affinity tag, with modified multiple cloning sites	[49]
pET28aNS-*Ca*BglA	pET28aNS derivative for expression of *Ca*BglA	This work
pET28aNS-*Ct*BglA	pET28aNS derivative for expression of *Ct*BglA	This work
pET28aNS-CglT	pET28aNS derivative for expression of CglT	This work
pET28aNS-Td2f2	pET28aNS derivative for expression of Td2f2	This work

### Construction of plasmids

Four recombinant plasmids, pET28aNS-*Ca*BglA, pET28aNS-*Ct*BglA, pET28aNS-CglT, and pET28aNS-Td2f2 (Table 2), were constructed for BGL expression in *E. coli* by cloning the genes *caBglA* (GenBank accession number: JX030398.1)*, ctBglA* (GenBank accession number: WP_003520797.1)*, cglT* (GenBank accession number: Z56279.1), and *td2f2* (GenBank accession number: HV538882.1) into the vector pET28aNS [49], respectively. The genes *caBglA, ctBglA*, and *cglT* were obtained by PCR with the genome DNAs of *Caldicellulosiruptor* sp. F32, *C. thermocellum* DSM1313 and *T. brockii* DSM1457 as the templates using primers *Ca*BglA-F/R, *Ct*BglA-F/R and CglT-F/R (Additional file 9), respectively. The gene *td2f2* was synthesized (Sangon, Beijing, China) with optimized encoding sequence to adapt the codon usage of *E. coli* [50]. Codon-modified *caBglA* gene, designated as *caBglAm* (GenBank accession number: KY418041), was synthesized based on the codon usage bias of *C. thermocellum* (Sangon, Beijing, China).

All plasmids constructed for the genetic manipulation in *C. thermocellum* were derived from pHK (GenBank accession number: KY792637) [51]. The construct the plasmid pHK-∆pyrF, ~1-kb upstream and downstream homologous arms of *pyrF* were amplified from the genome DNA of *C. thermocellum* DSM1313 using primer sets PyrF5′-F/R and PyrF3′-F/R (Additional file 9), respectively, and were ligated together by overlap PCR using primers PyrF5′-F/PyrF3′-R. The obtained PCR product was cloned to pHK vector through *Pst*I and *Nhe*I restriction sites to generate pHK-∆pyrF (Table 2).

The seamless editing plasmid pHK-HR contains two selection gene markers and three regions of homology (Additional file 2). The endogenous selection marker *pyrF* (Clo1313_1266) was driven by its native promoter [37]. The selection marker *tdk* (Teth514_0091) was obtained from *Thermoanaerobacter* sp. X514 [36], and was expressed under the control of a glyceraldehyde-3-phosphate dehydrogenase (*gapDH*) promoter from *C. thermocellum* DSM1313. Three regions of homology, HR-up, HR-short, and HR-down, were amplified from the genome DNA of *C. thermocellum* DSM1313 according to the genome editing demand, in which the sequence of HR-short was the same with the 3′ region of HR-up. BGL-encoding genes *ctBglA* and *caBglA* were chosen as the candidate DNA sequences to knock in, and were amplified from the genome DNAs of *C. thermocellum* DSM1313 and *Caldicellulosiruptor* sp. F32, respectively. The termination codons of the BGL-encoding genes were eliminated for fused protein expression. All fragments were cloned into the pHK vector sequentially. The Tdk expression cassette with the *gapDH* promoter was first cloned into pHK using *Nhe*I and *Xba*I sites. Subsequently, a fragment containing the upstream homology HR-up, the PyrF cassette as well as the short homology HR-short was obtained by overlap PCR and cloned into the plasmid using *Xba*I and *Eag*I sites. The downstream homology HR-Down was then ligated into the plasmid using *Mlu*I and *Bam*HI sites. We determined to integrate BGL sequences between the catalyzing module and dockerin module of CelS on the chromosome. To maintain the individual functions of the adjacent modules, repeated GGT sequence encoding glycine residuals were introduced in the upstream (3′ end of HR-short) and downstream (5′ end of HR-down) of the target DNA sequence. Finally, *ctBglA, caBglA* or *caBglAm* gene sequence was cloned into the plasmid via MluI and EagI sites, resulting in the plasmids pHK-HR-*Ct*BglA, pHK-HR-*Ca*BglA and pHK-HR-*Ca*BglAm (Table 2), respectively, for seamless genome editing.

For the plasmid-dependent expressions of *Ct*BglA and *Ca*BglA in *C. thermocellum* DSM1313, the predicted promoter and signal peptide region of CelS in *C. thermocellum* DSM1313 was amplified with primer set Pcs-sigF/R (Additional file 9), and cloned into pHK-HR-*Ct*BglA and pHK-HR-*Ca*BglA using NheI and EagI sites to construct pHK-*Ct*BglA-Doc and pHK-*Ca*BglA-Doc (Table 2), respectively. The Tdk cassette, HR-Up region, PyrF cassette, and HR-short region of the plasmids were replaced by the promoter and signal peptide of CelS. Thus, the BGL-encoding genes would be driven by the CelS promoter, and the expressed proteins would contain the signal peptide of CelS for protein secretion. Because the HR-Down region contained the encoding sequence of the dockerin module of CelS, the expressed BGLs would bear the dockerin module for cellulosome assembly.

### Heterologous expression and purification of beta-glucosidases in *E. coli*

The plasmids pET28aNS-*Ca*BglA, pET28aNS-*Ct*BglA, pET28aNS-CglT, and pET28aNS-Td2f2 were transformed into *E. coli* BL21(DE3) for heterologous expression of *Ca*BglA, *Ct*BglA, CglT, and Td2f2, respectively. Synthesis of recombinant proteins in *E. coli* BL21(DE3) cells was initiated by the addition of 1 mM IPTG, and cultivation was continued for an additional 16 h at 16 °C. Cells were harvested by centrifugation at 10,000 rpm, resuspended in 50 mM Tris–HCl buffer containing 30 mM imidazole and 300 mM NaCl, pH 8.0, and lysed by ultrasonication. The supernatants were applied onto a Histrap™ HP Ni-affinity column (GE Healthcare). The proteins were eluted with 50 mM Tris–HCl buffer containing 500 mM imidazole and 300 mM NaCl, pH 8.0. The eluted fractions were then concentrated to 2 mL using Amicon Ultra-15 centrifugal filter units (10.0 kDa cutoff) (Merck Millipore, Billerica, MA, USA), and applied onto a Superdex 75 gel filtration column (GE Healthcare) with 50 mM K_2_HPO_4_–KH_2_PO_4_ buffer with 100 mM KCl, pH 6.0.

### Preparation of cellulosomal and extracellular proteins

*Clostridium thermocellum* strains were cultivated in GS-2 medium with 5 g/L Avicel as the sole carbon source at 55 °C for 48 h. 200-mL cultures were centrifuged at 3000*g* for 30 min, the cell pellets were washed twice and resuspended in 4 mL 50 mM Tris–HCl buffer containing 5 mM DTT, pH 7.0, and lysed using a high-pressure homogenizer (Constant Systems LTD). The extracellular proteins were prepared by condensing 10 mL of the culture supernatants to 0.5 mL using Amicon Ultra-15 centrifugal filter units (10.0 kDa cutoff) (Merck Millipore, Billerica, MA, USA). The rest of the culture supernatants were used for cellulosome extraction according to a modified cellulose affinity procedure [10].

### Protein analyses

Sodium dodecyl sulfate–polyacrylamide gel electrophoresis (SDS-PAGE) was performed to check the protein purity and composition as previously described [10]. The molecular weight of the protein was estimated according to the relative mobility of protein ladders (10–245 kDa, New England BioLabs). The Bradford method was used for protein quantification [52]. The mass spectroscopy analyses were achieved according to a published procedure [10]. All protein samples were stored at −80 °C for further analyses.

### Enzyme assay

The BGL activity was determined against *p*-nitrophenyl-β-d-glucopyranoside (*p*NPG). Samples were incubated in 200-μL reaction buffer (50 mM sodium acetate, 1 mM *p*NPG, pH 5.5) at 55 °C for 5 or 10 min. The reaction was terminated by adding 1 mL of 1 M Na_2_CO_3_, and the absorbance of the mixture was measured at 405 nm immediately. One unit of enzyme activity was defined as the amount of enzyme required to produce 1 μmol of *p*-nitrophenol (*p*NP) per min under certain conditions.

The cellulase activity was tested in 1-mL final reaction volume containing 50 μg cellulosome proteins and 15 mg Avicel or 7.5 mg cellobiose as a substrate. The reaction buffer contained 50 mM sodium acetate, 10 mM CaCl_2_ and 5 mM dithiothreitol (DTT), pH 5.5. The abundance of reducing sugars was determined by the 3,5-dinitrosalicylic acid (DNS) method after incubation at 55 °C for 24 h, and the glucose and cellobiose in the hydrolysates were quantified by high-performance liquid chromatography (HPLC) as previously described [53].

### Electrotransformation and screening of *C. thermocellum*

The pHK derivative plasmids were transformed into *E. coli* BL21(DE3) to remove Dcm methylation, and transformed to *C. thermocellum* DSM1313 according to published protocols [10, 51]. In brief, 200 μL of *C. thermocellum* competent cells was added to 0.2-cm electroporation cuvettes (BioRad) with 10 μL of DNA (~2000 ng) in sterile distilled water. A series of 40 square pulses were applied, each with an amplitude of 1.5 kV and for a duration of 50 s at 500-ms intervals. Cells were then recovered for 24 h at 51 °C in 4 mL of fresh GS-2 medium before screening on solid medium containing Tm. The ∆*pyrF* mutant was selected as described [37]. In detail, the transformants containing pHK-∆pyrF were cultivated with the presence of Tm and then plated in GS-2 solid medium with 500 μg/mL FOA. FOA-resistant colonies were screened by colony PCR using PyrF-F/R flanking the genomic gene *pyrF*. The colonies with 0.25-Kb PCR products were determined as *pyrF*-deleted mutants.

The mutant ∆*pyrF* was then used as the parent to construct other *C. thermocellum* recombinant strains. The ∆*pyrF* transformants containing pHK-derived plasmids were initially selected on solid GS-2 medium supplemented with Tm, and then inoculated into liquid GS-2 medium with Tm to strengthen the replication of the transformed plasmid before further screening. Transformants containing plasmid pHK-*Ct*BglA-Doc or pHK-*Ca*BglA-Doc were verified by detecting the *Ct*BglA-Doc or *Ca*BglA-Doc fragments via colony PCR and sequencing. Transformants containing pHK-HR-*Ct*BglA, pHK-HR-*Ca*BglA or pHK-HR-*Ca*BglAm were further inoculated into MJ medium containing FUDR and cultivated until the late exponential phase. 200-μL cultures were diluted for 1, 10, 100, and 1000 fold, plated in MJ solid medium containing FUDR, and then cultivated at 51 °C for 5–7 days. The obtained colonies were screened by colony PCR using primer set HR-F/R (Additional file 9). Those colonies showing the PCR product of ~5.7 Kb indicated the first round of recombination was done via the long regions of homology. A band of 2.9 Kb might also be detected, indicating the mixing of the parent strain ∆*pyrF* (Additional file 10). If so, the FUDR screening in MJ medium should be repeated until single band of ~5.7 Kb was detected. The verified colonies were inoculated into GS-2 liquid medium with Tm to confirm the plasmid curing if no growth was observed, and further cultivated in GS-2 medium containing FOA to the late exponential phase, diluted, and plated in solid medium with FOA to select colonies without PyrF function. Colony PCRs were subsequently performed using primer set HR-F/R to confirm *pyrF* elimination. The colonies with PCR product of 4.7 Kb were finally verified as the target strain after sequencing.

### Fermentation of *C. thermocellum* strains

Fermentation of *C. thermocellum* strains were in 250-mL anaerobic bottles containing 100-mL cultures using 10 g/L Avicel as the sole carbon source. Three independent fermentations were set up for each strain, and 1.5-mL cultures were sampled every 8 to 12 h with a 2.5-mL syringe. 1 mL of each sample was used to determine the amount of residual cellulose by a modified saccharification method [10], and 0.5 mL of each sample was centrifuged for pellet cells to determine the abundance of total cellular protein [10].

### Cellulose saccharification

100-mL fermentations of *C. thermocellum* strains were initially performed with 5 g/L Avicel as the sole carbon source for 36 h. For aerobic treatment, the cultures were transferred into 250-mL sterile flasks shaking at 170 rpm aerobically. For acidic treatment, the pH value of the broths was adjusted to 5.5 by adding 1 N HCl in an anaerobic chamber. For BGL treatment, 15 U/g cellulose of *Ca*BglA protein was added at the beginning of the saccharification process. For high-intensity treatment, cells from 200- to 300-mL culture were concentrated, resuspended, and reinoculated into 100-mL fresh GS-2 medium anaerobically. Untreated controls were prepared under consistent conditions. For all setups, 100 g/L Avicel was supplemented to initiate the cellulose-saccharification stage. The saccharification process lasted for 15–20 days, and 1-mL sample was taken from each setup with a 5-mL syringe per 1 to 5 days to determine the abundance of produced reducing sugar by the DNS method, and the concentrations of sugars (cellobiose and glucose) and other metabolites (pyruvate, ethanol, lactate, acetate and formate) by HPLC. The relative saccharification level was determined subsequently by dividing the initial Avicel (617 mM glucose equivalents) with the amounts of the obtained reducing sugar (mM). Three independent experiments were prepared for every strain under each condition.

## Additional files

**Additional file 1: Figure S1.** The optimal temperature (a), pH value (b), and glucose inhibition (c) of *Ca*BglA and *Ct*BglA. The optimal temperature was determined by incubate the reaction mixture in 50 to 80 °C water bath for 10 min. The pH value of the reaction buffer was adjusted from 4.0 to 8.0 to determine the optimal pH. The glucose inhibition was determined by adding 0 to 400 mM glucose to the standard reaction mixture, and calculated as the glucose concentration required to inhibit 50% of initial activity. Values are average ± standard deviation based on three independent replicates.

**Additional file 2: Figure S2.** Map of plasmid pHK-HR used for seamless genome editing in *C. thermocellum*. The plasmid is derived from a *E. coli/C. thermocellum* shuttle vector pHK. To construct pHK-HR plasmids, the Tdk expression cassette (gapDH-F/tdk-R), the fragment containing the upstream homology HR-up (HR-up-F/R), the PyrF expression cassette (HR-pyrF-F/R), the short homology HR-short (HR-short-F/R), the downstream homology HR-Down (HR-down-F/R), and the BGL genes are ligated into the plasmid substantially.

**Additional file 3: Figure S3.** Identification of fusion protein Cel-BGL-Doc peptides in the parent and *ΔpyrF*::*Ca*BglA strains by mass spectroscopy analysis. Cellulosomal and extracellular proteins with the size of ~135 kDa or ~75 kDa were investigated. The green highlights indicate peptides detected by mass spectroscopy. The amino acid sequences shown in black, purple, and blue belong to Cel (the catalyzing module of CelS), BGL (*Ca*BglA), and Doc (the assembling module of CelS), respectively. The linker sequences are shown in bold. The insertion sites of the BGL sequence are indicated by red triangles. *Ca*BglA sequences are detected in ~135-kDa but not ~75-kDa cellulosomal and extracellular proteins of *∆pyrF*::*Ca*BglA, indicating the successful expression, secretion, and cellulosomal assembly of the protein Cel-*Ca*BglA-Doc in *ΔpyrF*::*Ca*BglA.

**Additional file 4: Figure S4.** Growth and fermentation analysis of *C. thermocellum* strains with Avicel as a carbon source. a, cell growth represented by the abundance of total protein in cell pellets. b, Avicel consumption in mM glucose equivalents. Average values and standard deviations are calculated based on three replicates for each strain.

**Additional file 5: Figure S5.** Production of reducing sugars by *∆pyrF*::*Ca*BglA. The concentration of produced reducing sugar was determined by DNS method. 0 or 15 U/g cellulose of *Ca*BglA were added. Three independent replicates were prepared to calculate the average values and standard deviations.

**Additional file 6: Figure S6.** Production of reducing sugars by *∆pyrF*::*Ca*BglA and *∆pyrF*::*Ca*BglAm. The concentration of reducing sugar was determined by DNS method. Three independent replicates were prepared to calculate the average values and standard deviations.

**Additional file 7: Figure S7.** Cell growth of *C. thermocellum ∆pyrF*::*Ca*BglA in GS-2 media containing different carbon and nitrogen sources. 10 or 20 g/L cellobiose and 1.3 g/L ammonium sulfate were supplemented when required. Regular GS-2 medium was used as control. Values are average ± standard deviation based on three independent replicates.

**Additional file 8: Figure S8.** Production of pellet and extracellular proteins by *∆pyrF*::*Ca*BglA with (pH 5.5) or without (Untreated) pH value adjustment at the cellulose hydrolysis stage. No dramatic change was detected during the whole saccharification process. But lower amounts of pellet and extracellular proteins were produced under pH 5.5 condition compared to the untreated control, indicating the blocked cell growth and the reduced expression of extracellular protein, including cellulosomal proteins. Two replicates were used for mean value calculation.

**Additional file 9: Table S1.** List of primers used in this study.

**Additional file 10: Figure S9.** Colony PCR screening of the *C. thermocellum* mutants after the first round of homologous recombination. Transformants of Δ*pyrF*::pHK-HR-*Ca*CglA grown on MJ solid medium with addition of FUDR were investigated using primer set HR-F/R. PCR product of ~5.7 Kb indicates the success of the first round of recombination, and a 2.9-Kb band refers to the parent strain ∆*pyrF* without genomic integration. Black arrows indicate the colonies showing double bands. The colonies contain both the parent strain ∆*pyrF* and the recombinant strain. M, DNA standards.
