# Supplementary figures and images for: Efficient whole-cell-catalyzing cellulose saccharification using engineered Clostridium thermocellum
Source: Biotechnol Biofuels. 2017 May 12;10:124. doi: 10.1186/s13068-017-0796-y (PMC5429504; doi:10.1186/s13068-017-0796-y)

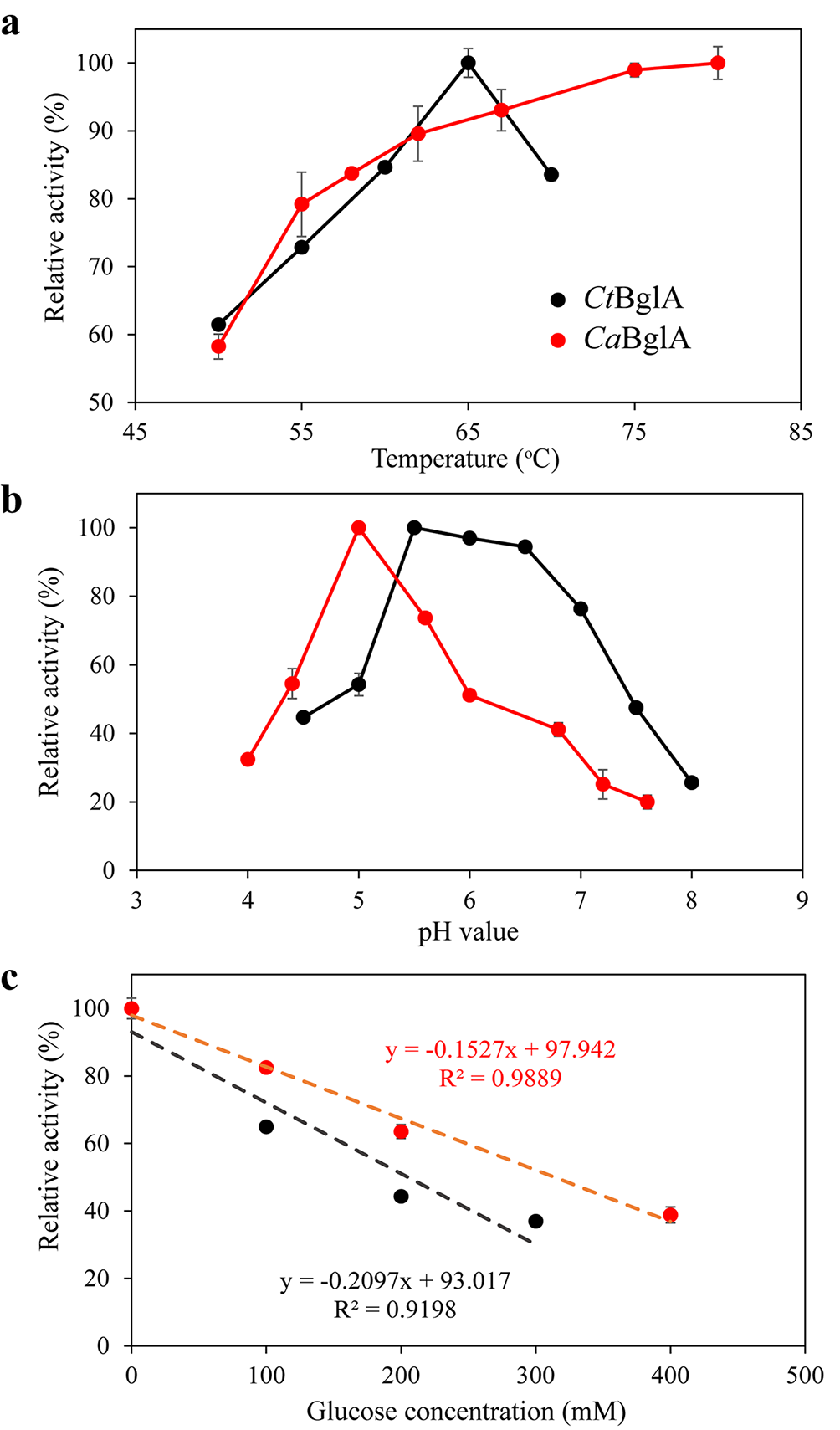

Supplement: Supplementary file 1 — Additional file 1: Figure S1. The optimal temperature (a), pH value (b), and glucose inhibition (c) of CaBglA and CtBglA. The optimal temperature was determined by incubate the reaction mixture in 50 to 80 °C water bath for 10 min. The pH value of the reaction buffer was adjusted from 4.0 to 8.0 to determine the optimal pH. The glucose inhibition was determined by adding 0 to 400 mM glucose to the standard reaction mixture, and calculated as the glucose concentration required to inhibit 50% of initial activity. Values are average ± standard deviation based on three independent replicates. [file 13068_2017_796_MOESM1_ESM.tif]

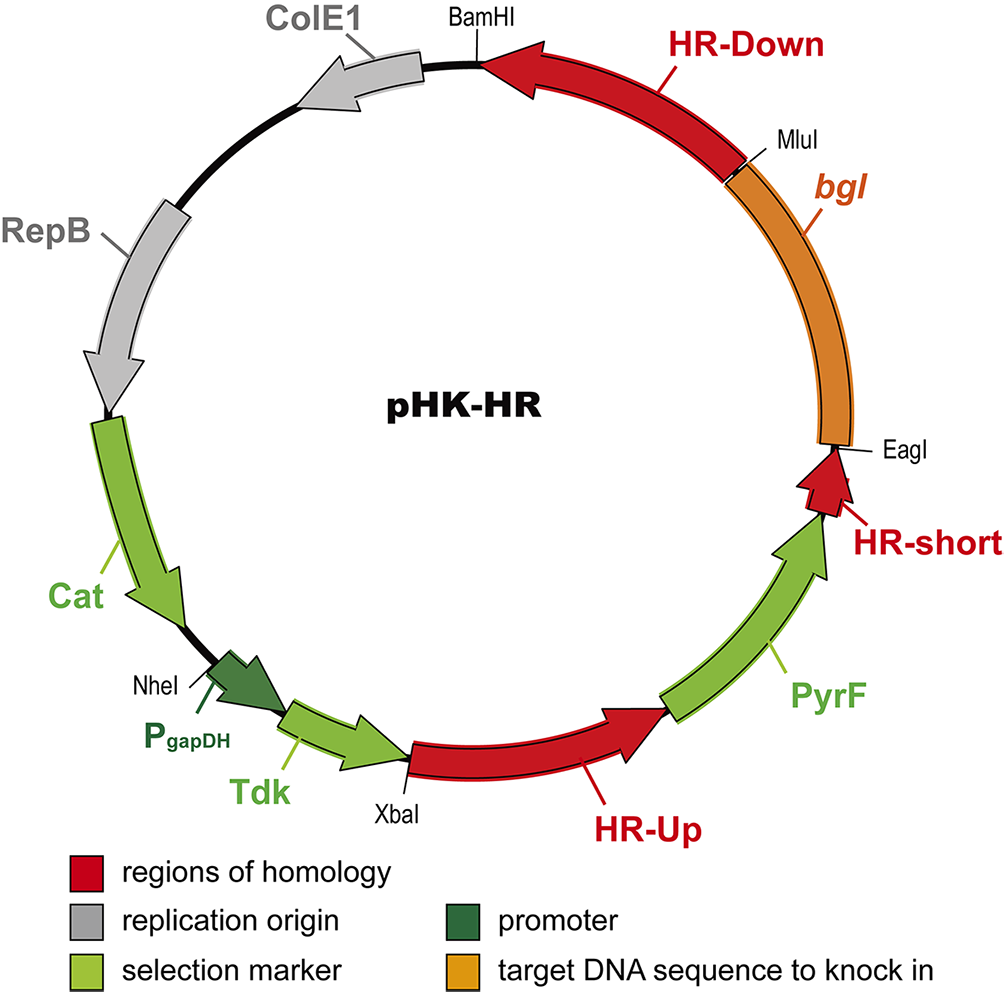

Supplement: Supplementary file 2 — Additional file 2: Figure S2. Map of plasmid pHK-HR used for seamless genome editing in C. thermocellum. The plasmid is derived from a E. coli/C. thermocellum shuttle vector pHK. To construct pHK-HR plasmids, the Tdk expression cassette (gapDH-F/tdk-R), the fragment containing the upstream homology HR-up (HR-up-F/R), the PyrF expression cassette (HR-pyrF-F/R), the short homology HR-short (HR-short-F/R), the downstream homology HR-Down (HR-down-F/R), and the BGL genes are ligated into the plasmid substantially. [file 13068_2017_796_MOESM2_ESM.tif]

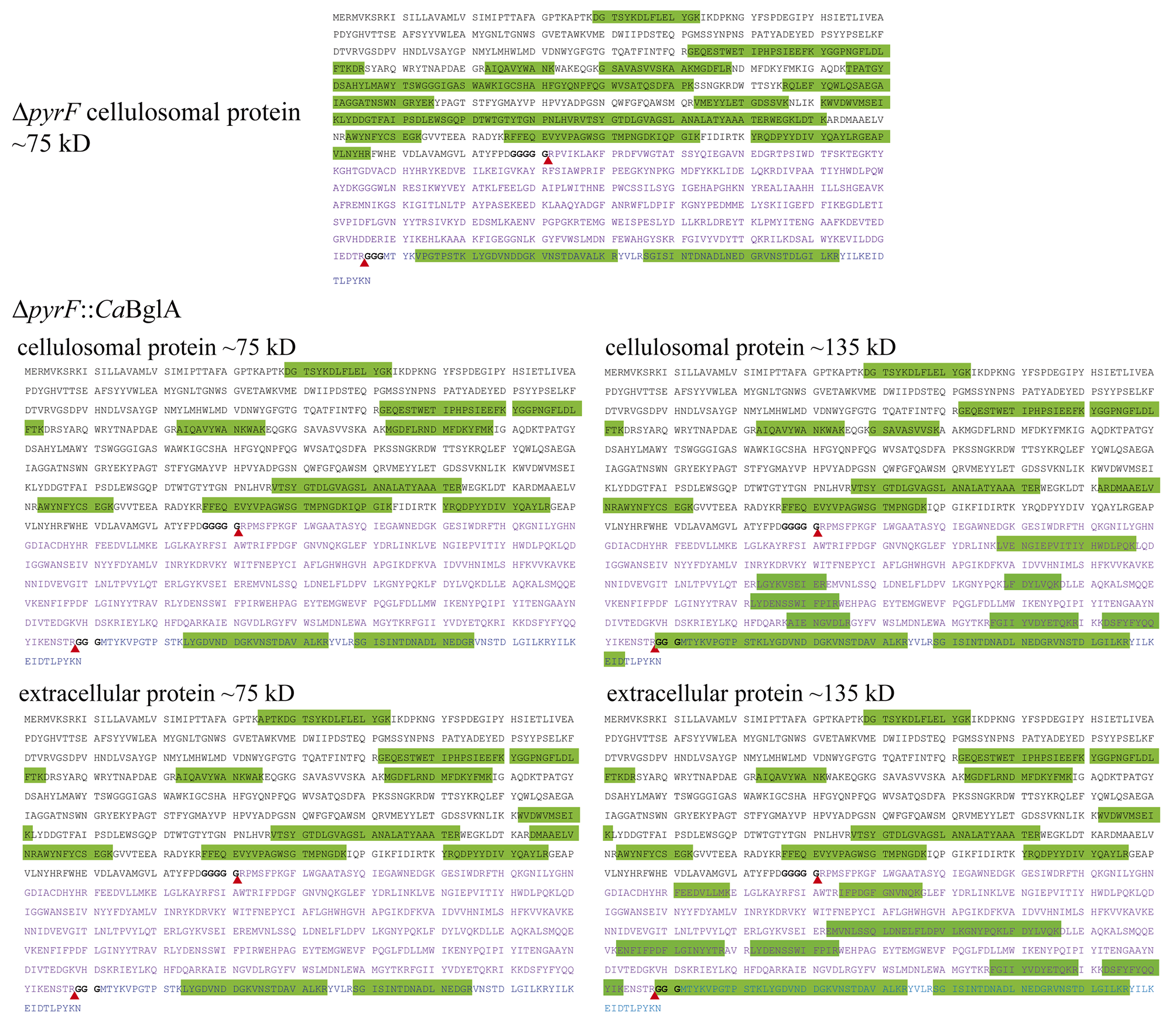

Supplement: Supplementary file 3 — Additional file 3: Figure S3. Identification of fusion protein Cel-BGL-Doc peptides in the parent and ΔpyrF::CaBglA strains by mass spectroscopy analysis. Cellulosomal and extracellular proteins with the size of ~135 kDa or ~75 kDa were investigated. The green highlights indicate peptides detected by mass spectroscopy. The amino acid sequences shown in black, purple, and blue belong to Cel (the catalyzing module of CelS), BGL (CaBglA), and Doc (the assembling module of CelS), respectively. The linker sequences are shown in bold. The insertion sites of the BGL sequence are indicated by red triangles. CaBglA sequences are detected in ~135-kDa but not ~75-kDa cellulosomal and extracellular proteins of ∆pyrF::CaBglA, indicating the successful expression, secretion, and cellulosomal assembly of the protein Cel-CaBglA-Doc in ΔpyrF::CaBglA. [file 13068_2017_796_MOESM3_ESM.tif]

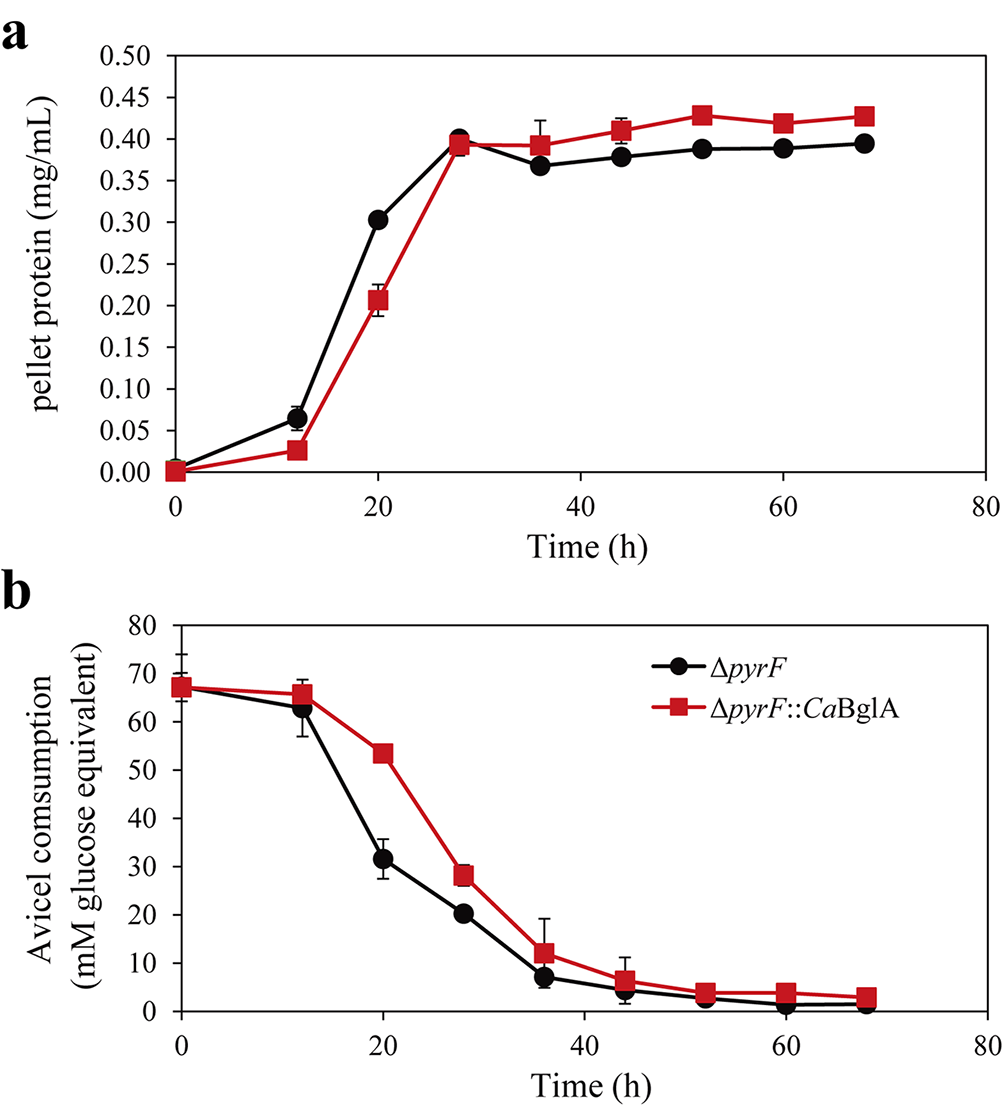

Supplement: Supplementary file 4 — Additional file 4: Figure S4. Growth and fermentation analysis of C. thermocellum strains with Avicel as a carbon source. a, cell growth represented by the abundance of total protein in cell pellets. b, Avicel consumption in mM glucose equivalents. Average values and standard deviations are calculated based on three replicates for each strain. [file 13068_2017_796_MOESM4_ESM.tif]

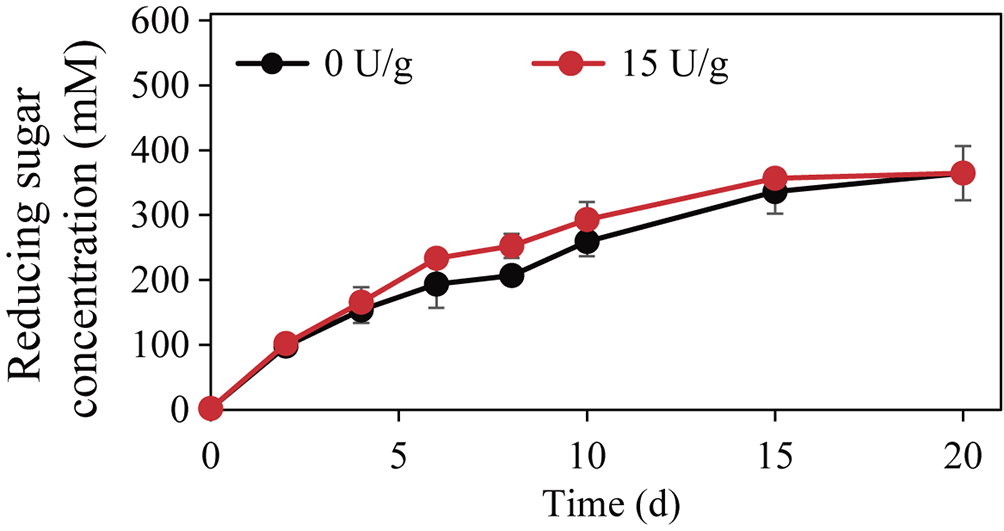

Supplement: Supplementary file 5 — Additional file 5: Figure S5. Production of reducing sugars by ∆pyrF::CaBglA. The concentration of produced reducing sugar was determined by DNS method. 0 or 15 U/g cellulose of CaBglA were added. Three independent replicates were prepared to calculate the average values and standard deviations. [file 13068_2017_796_MOESM5_ESM.tif]

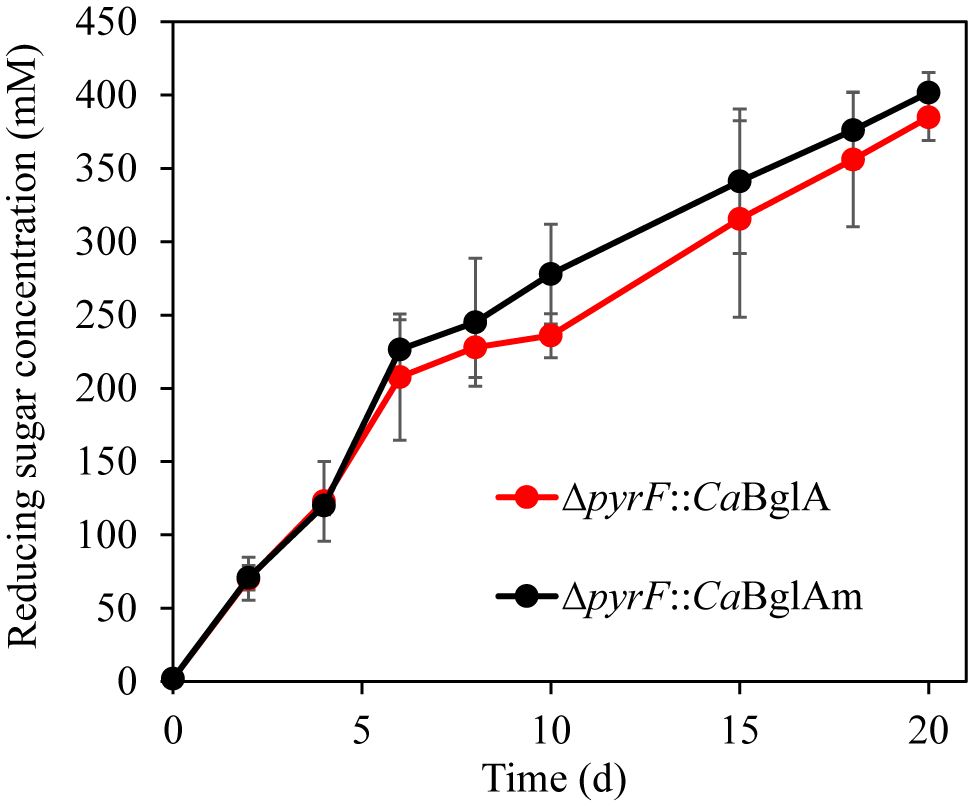

Supplement: Supplementary file 6 — Additional file 6: Figure S6. Production of reducing sugars by ∆pyrF::CaBglA and ∆pyrF::CaBglAm. The concentration of reducing sugar was determined by DNS method. Three independent replicates were prepared to calculate the average values and standard deviations. [file 13068_2017_796_MOESM6_ESM.tif]

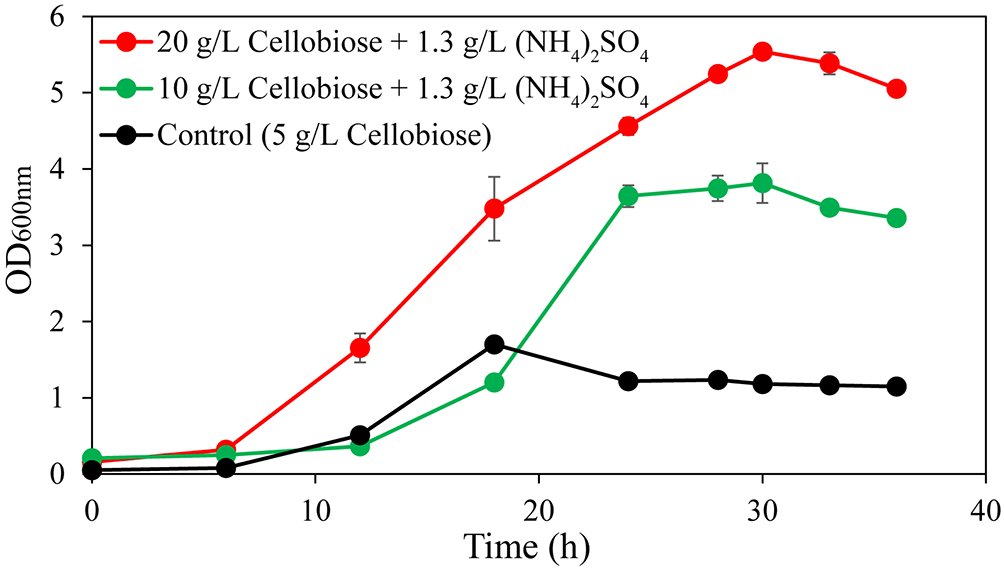

Supplement: Supplementary file 7 — Additional file 7: Figure S7. Cell growth of C. thermocellum ∆pyrF::CaBglA in GS-2 media containing different carbon and nitrogen sources. 10 or 20 g/L cellobiose and 1.3 g/L ammonium sulfate were supplemented when required. Regular GS-2 medium was used as control. Values are average ± standard deviation based on three independent replicates. [file 13068_2017_796_MOESM7_ESM.tif]

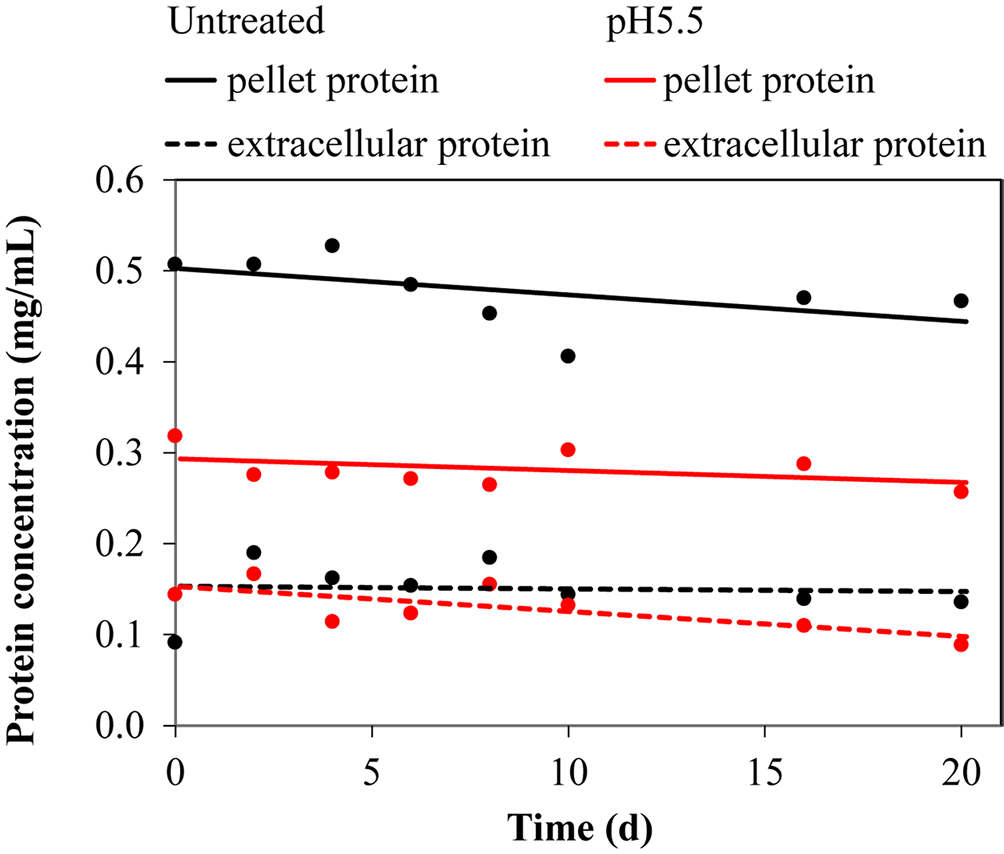

Supplement: Supplementary file 8 — Additional file 8: Figure S8. Production of pellet and extracellular proteins by ∆pyrF::CaBglA with (pH 5.5) or without (Untreated) pH value adjustment at the cellulose hydrolysis stage. No dramatic change was detected during the whole saccharification process. But lower amounts of pellet and extracellular proteins were produced under pH 5.5 condition compared to the untreated control, indicating the blocked cell growth and the reduced expression of extracellular protein, including cellulosomal proteins. Two replicates were used for mean value calculation. [file 13068_2017_796_MOESM8_ESM.tif]

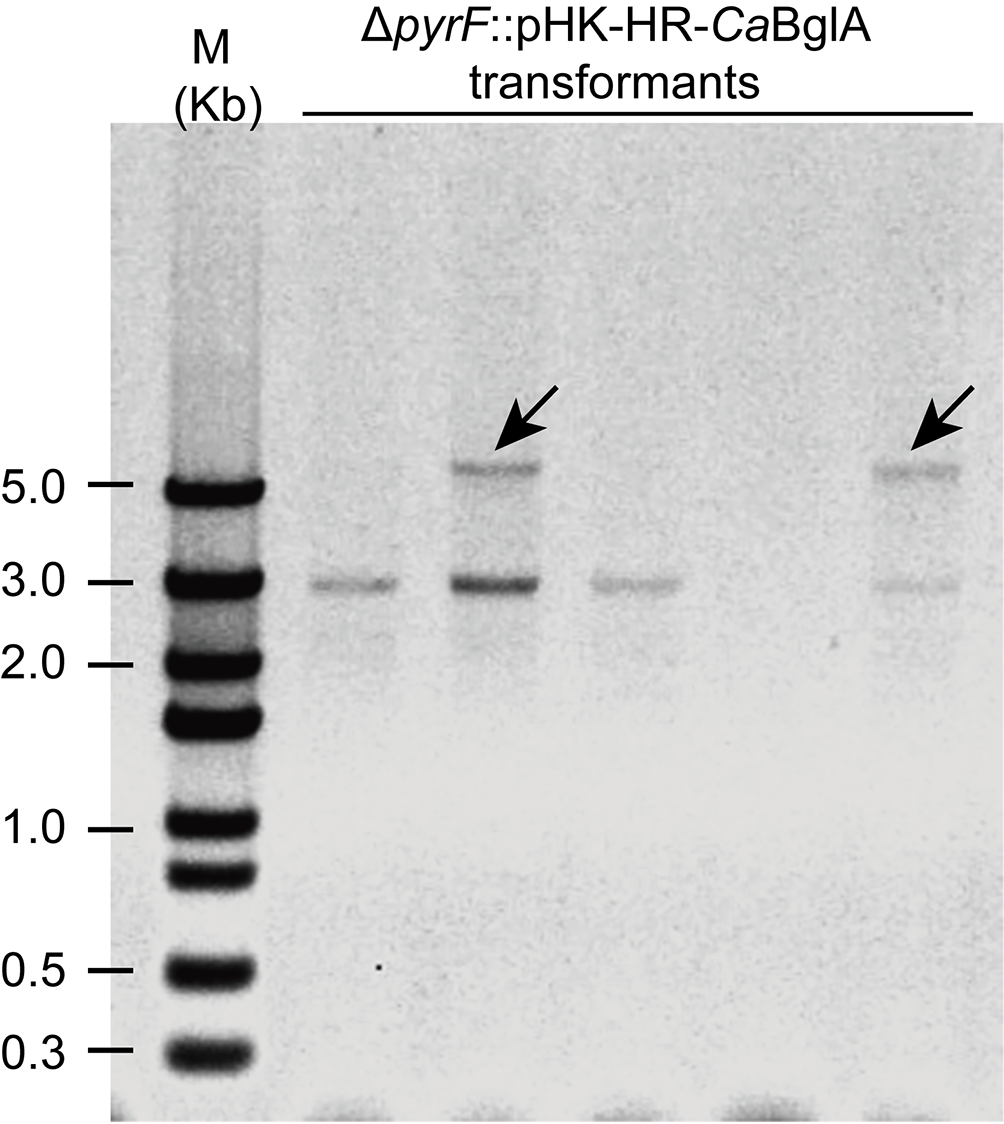

Supplement: Supplementary file 10 — Additional file 10: Figure S9. Colony PCR screening of the C. thermocellum mutants after the first round of homologous recombination. Transformants of ΔpyrF::pHK-HR-CaCglA grown on MJ solid medium with addition of FUDR were investigated using primer set HR-F/R. PCR product of ~5.7 Kb indicates the success of the first round of recombination, and a 2.9-Kb band refers to the parent strain ∆pyrF without genomic integration. Black arrows indicate the colonies showing double bands. The colonies contain both the parent strain ∆pyrF and the recombinant strain. M, DNA standards. [file 13068_2017_796_MOESM10_ESM.tif]
