# Supplementary material for: Efficient whole-cell-catalyzing cellulose saccharification using engineered Clostridium thermocellum
Source: Biotechnol Biofuels. 2017 May 12;10:124. doi: 10.1186/s13068-017-0796-y (PMC5429504; doi:10.1186/s13068-017-0796-y)
Supplement: Supplementary file 9 — Additional file 9: Table S1. List of primers used in this study. [file 13068_2017_796_MOESM9_ESM.docx]

Table S1. List of primers used in this study

| Primers | Sequences (5’-3’)^*^ |
| --- | --- |
| *Ca*BglA-F  *Ca*BglA-R  *Ct*BglA-F  *Ct*BglA-R  CglT-F  CglT-R  PyrF3`-F  PyrF3`-R  PyrF5`-F  PyrF5`-R  PyrF-F  PyrF-R  gapDH-F  gapDH-R  tdk-F  tdk-R  HR-up-F  HR-up-R  HR-pyrF-F  HR-pyrF-R  HR-short-F  HR-short-R  HR-down-F  HR-down-R  HR-CglT-F  HR-CglT-R  HR-F32BglA-F  HR-F32BglA-R  HR-CtBglA-F  HR-CtBglA-R  HR-F  HR-R  Pcs-sigF  Pcs-sigR | CTAGCTAGCATGATACTTTCTGAGCCT (NheI)  CCGCTCGAGTTATTCCCGAATAAAAGAAC (XhoI)  CTAGCTAGCATGTCAAAGATAACTTTCCCAAAAG (NheI)  CCGCTCGAGAAAACCGTTGTTTTTGATTAC (XhoI)  CTAGCTAGCGTGATAAAATTGGCAAAATTTC (NheI)  CCGCTCGAGTTAATCTTCGATACCATCAT (XhoI)  AAACTGCAGCATATGGACAGTGCCGTCGTTCATGTTT  AGGATTAGACCCTAAAATTGCGATATTAACAGTGCGTTGCGAG  CTCGCAACGCACTGTTAATATCGCAATTTTAGGGTCTAATCCT  CTAGGCGCCGCTAGCTAATATCCCATTGCGAAGAT  GTGTTATGTAAGGAGAATGA  TTACTTCCTGTCTCGCAACG  GGAATTCGCTAGCCTGGATACTTTGGAGGACGAGG (NheI)  GATCCAAAGATCCGTATATCATTAATATCGCCTCCTATTGTAAAT  ATTTACAATAGGAGGCGATATTAATGATATACGGATCTTTGGATC  GCTCTAGACTATGTACGGGGAACTTCATGG (XbaI)  GCTCTAGAcaaaggagcagggtaaaggttctg (XbaI)  GGTATAATATACACTTCCATTCAGatccgggaagtatgtagccaatac  GTATTGGCTACATACTTCCCGGATCTGAATGGAAGTGTATATTATACC  CAACACCTTTTCCTTCAGAGCAGTTACTTCCTGTCTCGCAACGCAC  GTGCGTTGCGAGACAGGAAGTAActgctctgaaggaaaaggtgttg  CACCGGCCGaccaccaccaccaccatccgggaagtatgtagccaatac (EagI)  GATACGCGTggtggtggtatgacatataaagtacctggtac (MluI)  CGGGATCCtgtacacattcgaatttgttacg (BamHI)  GTCGGCCGGTGATAAAATTGGCAAAATTTCC (EagI)  CCACGCGTATCTTCGATACCATCATCCAAT (MluI)  CCCGGCCGatgagtttcccaaaaggatt (EagI)  CCACGCGTCGAATTTTCCTTTATATACTGC (MluI)  AATTTACCCGGCCGATGTCAAAGATAACTTTCCC (EagI)  TCTGGGCCACGCGTAAAACCGTTGTTTTTGATTAC (MluI)  GGTACAGGAACACAGGCAACA  CATATTTGCGGCTCAGGCTAT  CGGCTAGCGGAGCGAATGCCCCGCTGAGC (NheI)  CTCGGCCGCCCATCTTTTGTAGGTGCCTTTG (EagI) |

* Restriction sites are underlined and indicated in following parentheses. Lowercase letters indicate linker sequences encoding three or five Glycine residuals.
